# Complete remission by transarterial infusion with cisplatin for recurrent bile duct tumor thrombus of hepatocellular carcinoma: report of a case

**DOI:** 10.1186/1477-7819-11-78

**Published:** 2013-03-23

**Authors:** Chiharu Ebara, Shintaro Yamazaki, Masamichi Moriguchi, Yusuke Mitsuka, Tomoya Funada, Tokio Higaki, Tadatoshi Takayama

**Affiliations:** 1 Department of Digestive Surgery, Nihon University School of Medicine, 30-1 Oyaguchikami-machi, Itabashi-ku, Tokyo, 173-8610, Japan

**Keywords:** Bile duct tumor thrombus, Hepatocellular carcinoma, Trans-arterial chemotherapy

## Abstract

Bile duct tumor thrombus (BDTT) of a hepatocellular carcinoma (HCC) is a rare entity which was found microscopically in 1 to 9.2% of the resected specimen.

A 54-year-old male was found to have a 65-mm hepatocellular carcinoma in segment VI of the liver with a huge intrahepatic bile duct tumor thrombus. As the main trunk of the posterior segment branched from the left bile duct, the BDTT of the posterior branch extended to the common bile duct via the left bile duct. When the posterior segment was resected along with the left lobe, the estimated remnant liver volume was less than 30%. Therefore, the patient underwent extended posterior segmentectomy with choledochotomy and all of the BDTT was removed via the common bile duct.

Three months later, his serum bilirubin (6.63 mg/dL) and des-gamma-carboxy prothrombin (410 ng/mL) were re-elevated due to recurrent BDTT. A well-enhanced BDTT was observed by computed tomography (CT) at the left bile duct. Transarterial chemotherapy with cisplatin was scheduled, followed by endoscopic retrograde bile duct drainage. After four sessions of this chemotherapy, the BDTT had vanished and the tumor marker was decreased to within the normal range. The patient was stably treated with this regimen and has remained recurrence-free for five years.

## Background

Bile duct tumor thrombus (BDTT) of a hepatocellular carcinoma (HCC) is a rare entity which was found microscopically in 1 to 9.2% of the resected specimen of this male patient. An icteric hepatoma was found obstructing the common bile duct (CBD). The prognosis for BDTT with palliative treatment was poor, about 3 to 13 months, and with no treatment was three months or less [1,2]. However, there was a chance for long time survival if the BDTT was surgically removed with the primary lesion [3,4].

The available treatment options for palliative treatment are transarterial chemoembolization, radiation and a combination of these modalities. But the treatment effects are limited because the tumor is usually wide-spread in the liver.

We experienced a case in which recurrent BDTT was put into complete remission by transarterial chemotherapy (TAC) with cisplatin. TAC with cisplatin may be one of the additional options following a palliative operation.

## Case report

**Figure 1:**
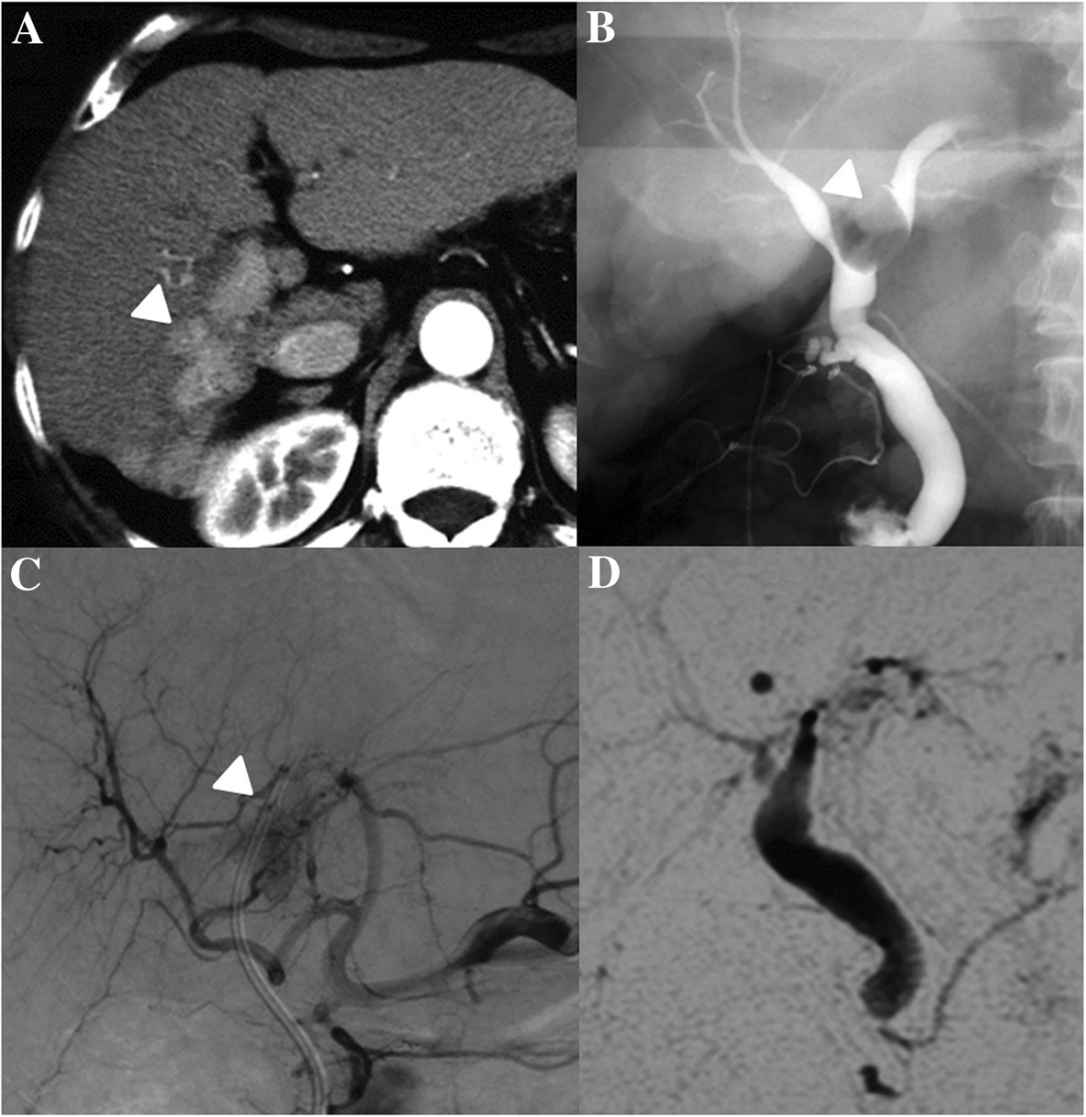
**(A) A well enhanced tumor thrombus of a HCC involving the portal vein and bile duct dilatation were found (arrow head).** (**B**) The intraoperative chorengiography revealed that bile duct tumor thrombus was extended from the posterior bile duct to the CBD via the left bile duct (arrow head). (**C**) The well enhanced recurrent bile duct tumor thrombus was found in the common bile duct (arrow head). (**D**) After a total of four treatments of TAC with cisplatin, the BDTT was observed with magnetic resonance cholangiopancreatography to be in complete remission.

A 55-year-old man was admitted to our department for treatment of a 65 mm HCC in segment VI of the liver. An HCC involving the portal vein and bile duct tumor thrombus were found on abdominal computed tomography (CT) (Figure 1A). The patient had severe liver cirrhosis due to a hepatitis B viral infection. The laboratory results showed that liver enzyme and serum alpha-fetoprotein (2.2 ng/mL) were within the normal ranges whereas des-gamma-carboxy prothrombin (DCP) (410 ng/mL) was increased.

During the operation, the intraoperative cholangiography confirmed that the posterior bile duct was branched from the left bile duct and the BDTT extended to the CBD via the left bile duct (Figure 1B). The remnant liver was less than 30% of the whole liver volume when the left lobe was resected along with the posterior segment. Therefore, the patient underwent extended resection of the posterior segment with choledochotomy to remove the BDTT in the left lobe. A massive infiltration of BDTT was found in the resected specimen.

Three months later, the patient was found to have obstacle jaundice due to recurrent BDTT. The serum bilirubin (6.63 mg/dL) and DCP (178 ng/mL) were increased in the laboratory data. Well enhanced BDTT in the arterial phase was confirmed with angiography (Figure 1C). Therefore, we planned trans-arterial chemotherapy followed by endoscopic retrograde bile duct drainage (Flexima, Boston Scientifics, Boston, MA, USA). A total of 70 mg/body of cisplatin powder (IA Call, Kaken, Tokyo, Japan) was infused through the common hepatic artery. After a total of four treatments with TAC with cisplatin, the BDTT was not observed on magnetic resonance cholangiopancreatography (Figure 1D) and the tumor markers were decreased to within normal range. TAC was discontinued but the patient remains in good condition without any new recurrence five years after the liver resection.

## Discussion

The median survival time in patients with BDTT is more than two to three years when the primary lesion with BDTT is completely resected [1-4]. In contrast, the median survival following the palliative operation is less than six months and with the biliary drainage alone is three months [5,6]. Thus, it is necessary to resect the primary lesion. However, most cases of BDTT are not good candidates for surgery because the primary lesion is advanced or BDTT is widely spread in the liver. Most cases present with liver insufficiency and contraindication for targeted therapy such as Sorafenib. While there are some treatment options for portal venous tumor thrombus caused by HCC, radiation and transarterial chemoembolization therapy enable the survival time to be extended in some studies [7,8].

TAC is an effective option for palliative treatment and median survival time was 13.4 months (range 8 to 26 months) [5,6]. The use of TAC with cisplatin in unresectable or recurrent HCC has been demonstrated recently [9-11]. This treatment regimen might be the best additional treatment for BDTT because the thrombus is fed by arterial blood [5-8]. Moreover, TAC enables to perform when the remnant liver was small or when patients have poor liver function. For our patient, we performed a total of four treatments of TAC with cisplatin and the BDTT subsided and tumor markers were decreased to normal. Due to the design of the study case series, we could not advocate TAC as a first treatment option in all BDTT cases.

The proposed treatment for BDTT is complete resection of the primary lesion with BDTT. However, our case suggested that TAC with cisplatin may have the potential to be an effective treatment option for BDTT in a palliative case.

## Conclusions

This case report suggested that transarterial chemotherapy with cisplatin may have the potential to be an effective treatment option for BDTT.

## Consent

Written informed consent was obtained from the patient for publication of this report and any accompanying images.

## Abbreviations

BDTT: Bile duct tumor thrombus; CBD: Common bile duct; CT: Computed tomography; DCP: Des-gamma-carboxy prothrombin; ERBD: Endoscopic retrograde bile duct drainage; HCC: Hepatocellular carcinoma; IHBD: Intrahepatic bile duct; TAC: Trans-arterial chemotherapy

## Competing interest

The authors declare that they have no competing interests.

## Authors’ contributions

CE drafted the manuscript, MM and YM performed TAC. TF and SY revised manuscript. TT designed this report. All authors read and approved the final manuscript.
